# Effect of nicastrin on hepatocellular carcinoma proliferation and apoptosis through PI3K/AKT signalling pathway modulation

**DOI:** 10.1186/s12935-020-01172-4

**Published:** 2020-03-24

**Authors:** Xicheng Wang, Xining Wang, Yunxiuxiu Xu, Maolin Yan, Wenxin Li, Jie Chen, Tao Chen

**Affiliations:** 1 Department of Hepatobiliary Surgery, Sun Yat-sen Memorial Hospital, Sun Yat-Sen University, Guangzhou, People’s Republic of China; 2 Guangdong Provincial Key Laboratory of Malignant Tumor Epigenetics and Gene Regulation, Medical Research Center, Sun Yat-sen Memorial Hospital, Sun Yat-sen University, Guangzhou, People’s Republic of China; 3 Department of Hepatobiliary Surgery, Fujian Provincial Hospital, Provincial Clinical College of Fujian Medical University, 134 East Street, Fuzhou, 350001 People’s Republic of China; 4 Department of Cardiology, The Eight Affiliated Hospital, Sun Yat-sen University, Shenzhen, People’s Republic of China

**Keywords:** NCSTN, Hepatocellular carcinoma, PI3K/AKT

## Abstract

**Background:**

Increasing evidence has proven that the γ-secretase complex plays significant roles in the carcinogenesis of malignancies. However, the independent effect of nicastrin (NCSTN), the largest constituent of the γ-secretase complex, on the progression of hepatocellular carcinoma (HCC) remains to be discovered.

**Methods:**

In our study, we used open online databases, including the Oncomine database, GEPIA and KMPlotter, to analyse the expression of 4 genes and their correlation with prognosis in HCC. NCSTN expression in 60 HCC patients from our centre was determined by immunohistochemical staining and qRT-PCR. The clinical and prognostic significance of NCSTN expression were analysed statistically. Stable Sk-hep1 cell lines with NCSTN overexpression were established using lentivirus-based vectors, and RNAi technology was used to transiently downregulate NCSTN expression in HepG2 cell lines. Cell growth and apoptosis were assessed by using EdU, clone formation, flow cytometry and Western blotting assays.

**Results:**

Bioinformatics analysis showed that NCSTN mRNA expression was generally higher in HCC tissues than in normal tissues according to a meta-analysis of 9 HCC datasets, excluding PS-1, PEN-2 and APH-1. Moreover, NCSTN was associated with a poor prognosis in HCC patients from The Cancer Genome Atlas (TCGA). Although the relationship between NCSTN levels and the clinicopathological features of HCC patients was not significant, a survival analysis of HCC patients from TCGA indicated that overall and disease-free survival were significantly associated with NCSTN expression. NCSTN expression in HCC cell lines regulated cell growth and apoptosis in vitro. NCSTN downregulation in HepG2 cells inhibited tumour growth ability in vivo. In addition, NCSTN downregulation in HepG2 cell lines decreased p-PI3K and p-Akt expression, and IGF1, a PI3K/Akt activator, neutralized the effects on PI3K and Akt phosphorylation. Moreover, NCSTN overexpression in Sk-hep1 cells activated the PI3K/Akt signalling pathway, and MK-2206, a PI3K/Akt inhibitor, reversed this activation according to Western blotting analysis.

**Conclusions:**

We suggest that NCSTN serves as an oncogene in HCC by promoting growth and inhibiting apoptosis via the PI3K/Akt pathway, providing a potential novel therapeutic target for HCC treatment.

## Background

Hepatocellular carcinoma (HCC) is one of the most common malignant cancers and is characterized by high mortality and a low 5-year survival rate [1]. Although curative therapies such as liver transplantation, radiofrequency ablation and radioembolization are rapidly developing and have improved early-stage HCC outcomes [2], the overall prognosis remains unsatisfactory because most patients are at an advanced stage when first diagnosed. Therefore, there is an unmet need for more effective treatments for advanced HCC. Accumulating evidence has demonstrated that the abnormal expression and mutation of genes are involved in the carcinogenesis and progression of HCC [3]. In this regard, therapies targeting a specific oncogenic molecule expressed in cancer tissue are a reliable approach for treating HCC. However, it is necessary to identify a potential therapeutic target to improve the clinical treatment outcome of HCC patients.

The γ-secretase complex (GS), composed of presenilin-1 (PS-1), anterior-pharynx defective-1 (APH-1), PSEN enhancer 2 (PEN-2) and nicastrin (NCSTN), can regulate endocytosis, lysosomal acidification, and autophagy [4, 5]. Dysfunction and abnormal activation of these substrates often leads to tumourigenesis, metastasis, and drug resistance in existing treatment regimens [6]. In recent years, the γ-secretase complex has been increasingly recognized as a vital target in human malignancies. NCSTN, one of the main constituents and the gatekeeper of the γ-secretase complex, modulates the composition of this complex, maintains enzyme stability and executes GS substrate recognition [7]. Moreover, NCSTN has a GS-independent function in the process of controlling cell death through the Akt pathway [8]. Consistent with the biological correlation of NCSTN in malignant diseases, studies have shown that NCSTN is upregulated in patients with colon cancer who underwent chemotherapy [9] and that stable knockdown of NCSTN enhances the antitumour effect of EGFR inhibitors by blocking the Notch and Akt signalling pathways [10]. Intriguingly, several studies have proven that genome-wide liver carcinoma screening identified NCSTN as one of the 50 potential driver genes [11], and NCSTN is abnormally upregulated and frequently amplified in liver cancer [12]. However, the precise relationship between NCSTN expression and HCC prognosis remains unknown. Together, these aforementioned studies prompted us to explore the unequivocal role of NCSTN in HCC progression and to confirm whether NCSTN could be a novel therapeutic target against HCC.

In this study, we hypothesized that NCSTN could activate the PI3K/Akt pathway to promote cell proliferation and inhibit cell apoptosis in HCC. To test this hypothesis, we used bioinformatics analyses to analyse the underlying function of NCSTN in malignant tumours. Gain-of-function and loss-of-function studies were utilized to validate the effects of NCSTN expression modulation in vitro and in vivo. Our work may be useful for developing a new therapeutic target for HCC.

## Methods

### Cell lines and culture

Seven HCC cell lines (Huh-7, Hep-3B, HepG2, PLC/PRF/5, Sk-hep1, 97H and SMMC-7721) were cultured in Dulbecco’s modified Eagle’s medium (DMEM; Gibco BRL, Grand Island, NY, USA) supplemented with 10% foetal bovine serum (FBS; Sijiqing, Zhejiang, P. R. China), 100 U/ml penicillin and 100 U/ml streptomycin. The HepG2, 97H, Sk-hep1, and SMMC-7721 cell lines were purchased from the American Type Culture Collection (Manassas, VA, USA). Huh-7, Hep-3B, and PLC/PRF/5 cell lines were purchased from the Cell Bank of the Chinese Academy of Sciences (Shanghai, P. R. China). All cells were maintained in a humidified chamber with 5% CO2 at 37 °C.

### Cell transfection

Three short hairpin (sh) RNAs targeting the CDS of human NCSTN mRNA were cloned into a lentiviral vector (PLKO.1), and the NCSTN open reading frame was cloned into a lentiviral vector (GENEray, Shanghai, China). HepG2 cells were first transfected with shNCSTN 1, shNCSTN 2 and shNCSTN 3 (40 nM) using Lipofectamine 2000 (Invitrogen; Thermo Fisher Scientific, Inc.) and were then infected with a lentivirus delivering shNCSTN 1 (sequence: CCGGGAGCATGCTAAAGCCTATAAACTCGAGTTTATAGGCTTTAGCATGCTCTTTTTG). A control plasmid (shNC) was used as a negative control. Meanwhile, lentiviruses carrying vector-NC or vector-NCSTN were produced from 293T cells and used to infect Sk-hep1 cells. After 72 h of lentiviral infection, puromycin (2 μg/ml) was used to select cells with stable NCSTN expression for 2 weeks. Selective NCSTN silencing in HepG2 cells and stable NCSTN expression in Sk-hep1 cells were detected by qRT-PCR and Western blotting analysis.

### Reagents and antibodies

Primary antibodies for proliferating cell nuclear antigen (PCNA) (#13110), p/t-PI3K (#4228/#4292), p/t-Akt (#4060/#4691), Bax/Bcl-2 (#5023/#4223), and caspase 3/cleaved caspase 3 (#9665) were purchased from Cell Signaling Technology (Danvers, MA, USA). An anti-NCSTN antibody (#14071-1-AP) was purchased from Proteintech (Chicago, USA). Antibodies against glyceraldehyde 3-phosphate dehydrogenase (GAPDH; #CW0100) and horseradish peroxidase (HRP)-labelled anti-rabbit secondary antibodies (#CW0102/#CW0103) were obtained from CWBIO (Beijing, P. R. China). MK-2206 (#SF2712) and recombinant human IGF-1 (#P5502) were purchased from Beyotime (Shanghai, China).

### Real-time polymerase chain reaction (qRT-PCR)

Total RNA was isolated from cells using TRIzol^®^ reagent (#15596-018; Invitrogen). Reverse transcription was performed using a PrimeScript RT reagent kit (#RR037; Takara) according to the manufacturer’s instructions. qRT-PCR was performed to evaluate NCSTN expression in different cell lines using the primers shown in Table 1. Relative gene expression levels were calculated using the comparative Ct (ΔΔCt) method, where the relative expression was calculated as 2-ΔΔCt, with Ct representing the threshold cycle.

**Table 1:** Primer sequences and their product lengths in qRT-PCR

Gene	Primer	Primer sequences	Length of product (bp)
PS-1	Forward	GACGACCCCAGGGTAACTC	151
	Reverse	ACTGACTTAATGGTAGCCACGA	
PEN-2	Forward	CTGGAGCGAGTGTCCAATGAG	171
	Reverse	GCGCCAGACATAGCCTTTGAT	
APH-1	Forward	TTTTTCGGCTGCACTTTCGTC	96
	Reverse	TGCGACCAGGATGATAACGC	
NCSTN	Forward	AATAAAACAGCTCCCTGTGTTCG	95
	Reverse	ACTACGTGGATAACCCCTGTG	
GAPDH	Forward	GGAGCGAGATCCCTCCAAAAT	197
	Reverse	GGCTGTTGTCATACTTCTCATGG	
U6	Forward	CTCGCTTCGGCAGCACA	94
	Reverse	AACGCTTCACGAATTTGCGT	

*PS-1* presenilin-1, *APH-1* anterior-pharynx defective-1, *PEN-2* PSEN enhancer 2, *NCSTN* nicastrin, *U6* U6 small nuclear 1 (RNU6-1)

### Immunohistochemistry (IHC)

After fixing with 4% formalin and embedding in paraffin wax, tissues were cut into 5-μm sections using a microtome. The sections were deparaffinized in xylene and rehydrated with graded concentrations of alcohol. Subsequently, the slides were treated with 3% H_2_O_2_ to block endogenous peroxidase activity, and antigen retrieval was performed by boiling in 1 mM EDTA (pH 8.0) for 10 to 15 min. To reduce nonspecific binding, 10% normal goat serum was applied. Then, the slides were incubated with a primary rabbit anti-human NCSTN antibody at 4 °C overnight. After washing with PBS, the sections were incubated with rabbit conjugate (#GK500705; Dako Corporation, Carpinteria, CA, USA) for approximately 1 h at 37 °C. The colour reaction was performed with DAB-positive substrate, and the slides were counterstained with haematoxylin. The staining intensity was classified into 4 grades: 0 (negative), 1 (weak), 2 (moderate), or 3 (strong). The percentage of NCSTN-positive cells was scored as 0 (0%), 1 (1–10%), 2 (11–50%), 3 (51–80%) and 4 (81–100%). The overall score was calculated using the following formula: overall score = intensity score x percentage score. Total scores of 0–4, 5–8, and 9–12 were defined as weak positive, moderate positive, and strong positive, respectively [13].

### Patients and specimens

HCC specimens and adjacent normal tissues were collected from 60 HCC patients at Sun Yat-sen Memorial Hospital of Sun Yat-sen University (Guangzhou, China) from 2016 to 2019. The specimens were immediately frozen in liquid nitrogen after resection from HCC patients and then stored at − 80 °C. The clinical characteristics of the HCC patients involved in the study are shown in Additional file 1: Table S2. The study was approved by the Ethics Committee of Sun Yat-sen Memorial Hospital. All patients provided informed consent for all treatments performed and to have their data used for research purposes.

### Western blotting analysis

To examine protein expression after shRNA and vector-NCSTN lentiviral transfection, HepG2 and Sk-hep1 cells were collected. Total protein was extracted from cells lysed with RIPA lysis buffer (#CW2333; CWBIO), phosphatase inhibitors (#04906845001; Roche) and protease inhibitors (#05892791001; Roche). Cells were lysed in 4× SDS sample buffer. Protein samples were then separated by SDS-PAGE (#P0012A; Beyotime) and transferred to polyvinylidene difluoride membranes (#ISEQ 00010; Millipore, Billerica, MA, USA). After blocking with 5% bovine serum albumin (BSA), the membranes were exposed to antibodies against NCSTN, PCNA, p/t-PI3K, p/t-Akt, Bax/Bcl-2, caspase 3/cleaved caspase 3, and GAPDH with continuous agitation overnight at 4 °C, followed by incubation with an anti-rabbit HRP-linked secondary antibody at room temperature for 1 h. The bands were visualized using an enhanced chemiluminescence detection kit (#WBKLS0500; Millipore).

### Cell viability assay

HepG2 cells were first transfected with shNCSTN 1, shNCSTN 2 and shNCSTN 3 (40 nM) using Lipofectamine 2000 (Invitrogen; Thermo Fisher Scientific, Inc.) Then, HepG2 cells were seeded into a 96-well plate at densities of approximately 1 × 10^4^ and 0.5 × 10^4^ cells/well. Next, the cells were cultured with medium containing 10% FBS for 48 h. Following the manufacturer’s protocol, a Cell Counting Kit-8 (CCK-8; #C0040; Beyotime) assay was utilized to quantify the viable cells. Absorbance (450 nm) was measured using a microplate reader (Dynex, Chantilly, VA, USA), and the absorbance value obtained from the responding control group was normalized as 100%.

### EdU assay

Cells were seeded in serum-free media 6 h prior to treatment to allow for cell cycle synchronization. After 48 h of shRNA interference or vector-NCSTN plasmid transfection, the cells were pulsed with 5-ethynyl-2′-deoxyuridine (BeyoClickTM EdU Cell Proliferation Kit with Alexa Fluor 488; #C0071; Beyotime) for 2 h before fixation in 4% paraformaldehyde (PFA) for 15 min and subsequent EdU detection per the manufacturer’s protocol. The results were imaged under an inverted fluorescence microscope. Quantification of EdU^+^ HCC cells was carried out using Adobe Photoshop CC 2018. The percentage of EdU^+^ cells for each field of view captured was recorded and analysed.

### Flow cytometry

Cells treated under different conditions for 48 h were detached from 6-well culture plates, washed twice with PBS, and pelleted by centrifugation at 1000 rpm for 5 min. The cell pellets were resuspended in binding buffer with Annexin V-FITC (AV) and propidium iodide (PI). The apoptosis rate was measured by flow cytometry.

### Colony formation assay

The effect of NCSTN knockdown on the colony-forming ability of HepG2 and NCSTN-overexpressing Sk-hep1 cells was analysed by colony formation assay. The infected cells were seeded into 6-well plates at a concentration of 400 cells per well. After incubation for 7 days, most single colonies contained more than 50 cells. The adherent cells were washed twice with PBS and then stained with crystal violet. Finally, the cells were rinsed with water, and pictures were taken.

### The cancer genome atlas (TCGA) database

The TCGA database contains gene information from large-scale genomic sequencing. Data including the gene expression profiles and clinical information of HCC patients were retrieved from the Genomic Data Commons Data Portal within the TCGA data portal (https://portal.gdc.cancer.gov/). The clinical information is shown in Additional file 2: Table S1, and the prognosis associated with NCSTN was calculated by several online databases, such as Oncomine (https://www.oncomine.org) [14], GEPIA (http://gepia.cancer-pku.cn) [15], KMPlotter (http://www.kmplot.com), CBioPortal (https://www.cbioportal.org) [16, 17], gene set enrichment analysis (GSEA) software [18] and String (https://string-db.org) [19], to obtain a more comprehensive understanding of the γ-secretase complex.

### Animal experiments

Female BALB/c nude mice, 4–7 weeks old, were purchased from the Laboratory Animal Center of Sun Yat-sen University (Guangzhou, China). The mice were maintained in a specific pathogen-free environment with laminar air-flow conditions at 22–25 °C. All animals had free access to standard laboratory mouse food and water. Animal experiments were approved by the Bioethics Committee of Sun Yat-sen University and were performed according to the established guidelines.

### Xenograft tumour growth

A total of 1 × 10^7^ HepG2 cells were suspended in 100 μL serum-free DMEM and injected subcutaneously into the nude mice. The mice were then randomized into two groups (n = 3/group). The tumour volume was estimated based upon the following formula: V = (L*W*W)/2, where “L” and “W” represent the larger and smaller dimensions obtained from each measurement, respectively. The nude mice were sacrificed after 1 month, and the solid tumours were weighed and fixed in formaldehyde.

### Statistical analysis

All data are represented as the mean ± SD from three independent experiments. The median expression level of NCSTN was used as the cutoff for the high NCSTN group and the low NCSTN group. Student’s *t* test was performed for statistical analysis, and *P* < 0.05 was considered significant.

## Results

### Bioinformatics analysis indicated that NCSTN was pivotal in promoting HCC progression

**Fig. 1:**
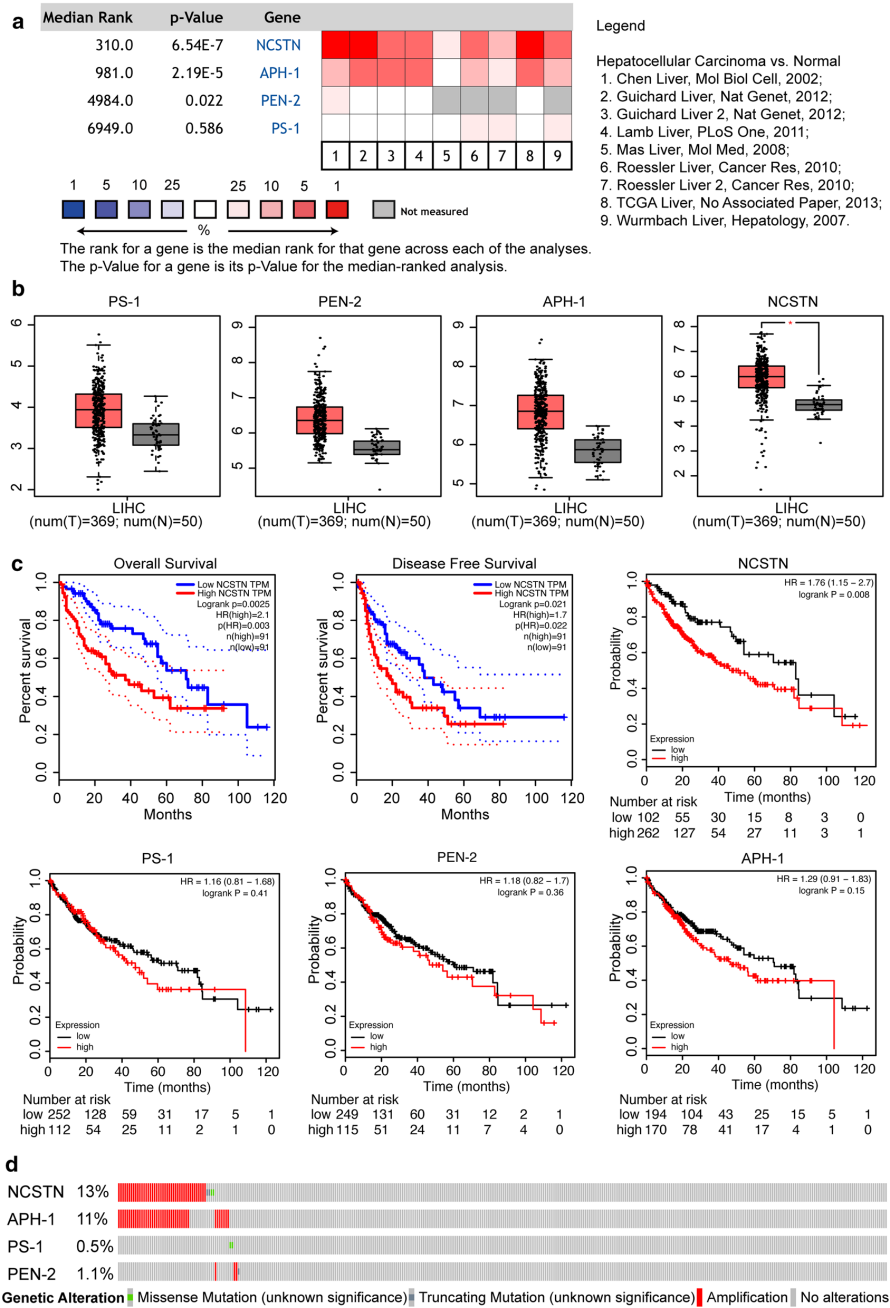
NCSTN is highly upregulated in hepatocellular carcinoma. **a** Meta-analysis of NCSTN, APH-1, PEN-2 and PS-1 in hepatocellular carcinoma based on the Oncomine database (http://www.oncomine.org). Nine databases comparing hepatocellular carcinoma tissues with corresponding normal tissues were used. The colour of the square represents the different levels of NCSTN expression. Red indicates overexpression in hepatocellular carcinoma, while blue indicates low expression. **b** Boxplot for NCSTN, APH-1, PEN-2 and PS-1 expression in HCC. The red and grey boxes represent liver tumour and normal tissues, respectively. The y-axis indicates the log2-transformed gene expression level. The data were obtained from TCGA through GEPIA (http://gepia.cancer-pku.cn). **c** High NCSTN expression in HCC indicated a poor prognosis. Overall survival and disease-free survival of HCC patients with high or low NCSTN expression in TCGA determined by GEPIA. Kaplan–Meier plots for NCSTN, APH-1, PEN-2 and PS-1 were generated by Kaplan–Meier Plotter (http://www.kmplot.com). **d** A visual summary displaying the genetic alteration of γ-secretase compounds in TCGA-LIHC patients, retrieved from the CBioPortal database (https://www.cbioportal.org)

We tried to determine whether NCSTN was the key gene in the progression of HCC patients by using bioinformatics analysis. As shown in Fig. 1a, a meta-analysis of 9 HCC datasets vividly showed that only NCSTN expression was higher in tumour specimens than in normal liver tissues over 9 datasets. However, only 7 datasets showed that APH-1 expression was slightly higher in tumour specimens. Moreover, 1 dataset revealed that PEN-2 expression was higher in tumour specimens, while 3 datasets showed that PS-1 had similar results. Moreover, these results were further confirmed in GEPIA, another online visualization site based on TCGA and GTEx data. The box plots shown in Fig. 1b reveal that NCSTN expression was markedly higher in liver hepatocellular carcinoma (LIHC) (*P *< 0.01). However, the other 3 members of the γ-secretase complex, excluding NCSTN, showed no obvious significance when comparing their expression between HCC and normal liver tissues (Fig. 1b). Importantly, the overall survival (OS) and disease-free survival (DFS) curves of LIHC patients with high or low NCSTN expression are shown in Fig. 1c. The results showed that not only the OS rate but also the DFS rate was significantly lower for patients with high NCSTN expression than for patients with low NCSTN expression. Furthermore, high NCSTN expression, but not the other 3 genes of the γ-secretase complex, indicated poor prognosis in 364 TCGA HCC patients, as analysed in the open online database KMPlotter (Fig. 1c). For genetic alterations, the CBioPortal database was used to analyse the changes in these four members using the mutation and CNA data of TCGA-LIHC patients. As shown in Fig. 1d, NCSTN was altered the most (13%), and the main type was amplification. In summary, bioinformatics analysis showed that high NCSTN expression represented a poor prognosis and may exert some underlying functions as an oncogene, independent of the γ-secretase complex.

### The NCSTN expression and prognosis of 60 HCC patients in our centre were significantly related

**Fig. 2:**
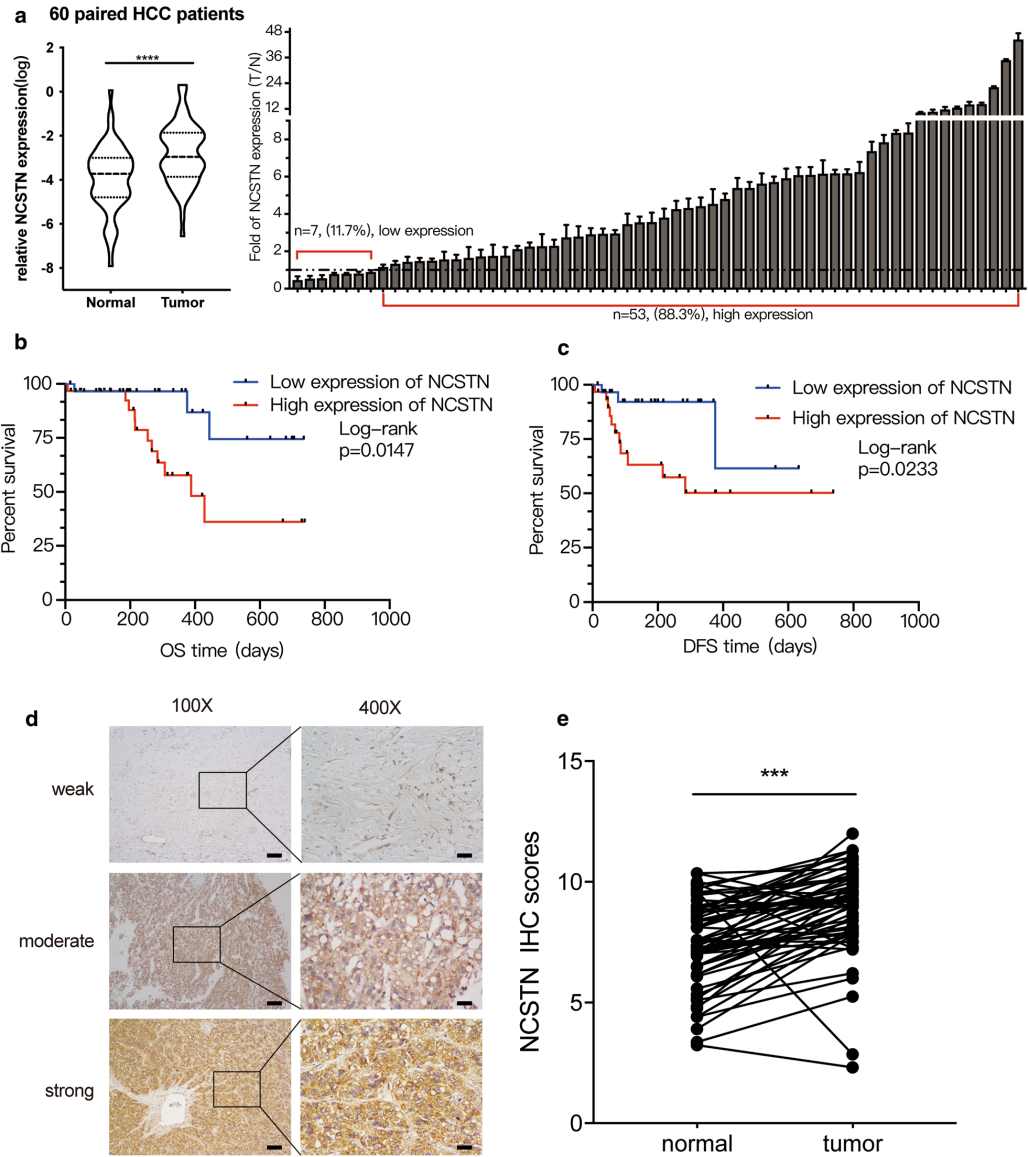
Correlation between NCSTN expression, OS and DFS in 60 HCC patients from our centre. **a** NCSTN mRNA expression in 60 HCC patients from our centre. The data represent the mean ± SD of three independent experiments. **b**, **c** NCSTN overexpression indicated poor OS and DFS. **d** IHC for NCSTN in 60 HCC patients. Scale bars: 400 μm (×100); 100 μm (×400). The ×100 scale bars represent 100 µm, and the ×400 scale bars represent 25 µm. **e** Paired NCSTN IHC scores for 60 patients (cancer versus normal tissues)

To analyse the mRNA expression levels of NCSTN in 60 HCC patients, we analysed matched tumour and normal tissues by using qRT-PCR. Overall, NCSTN expression was significantly upregulated in HCC. Compared with the normal tissues, 53 (88.3%) HCC patient tissues were characterized by higher NCSTN expression (Fig. 2a). Consistent with the bioinformatics analysis results, only 32 (53%) patients had high PS-1 expression, 38 (63.3%) patients had high PEN-2 expression, and 35 (58.3%) patients had high APH-1 expression (Additional file 3: Figure S1). In addition, by calculating the OS and DFS curves, we found that high NCSTN expression was related to poor prognosis in HCC patients (Fig. 2b, c). On the other hand, the IHC results of 60 HCC patients further confirmed that NCSTN expression was higher in tumour specimens than in matched normal tissues (Fig. 2d, e). Taken together, these results showed that high NCSTN expression was correlated with poor prognosis in HCC patients.

### Oncogenic function validated by NCSTN upregulation in Sk-hep1 cells and NCSTN downregulation in HepG2 cells

**Fig. 3:**
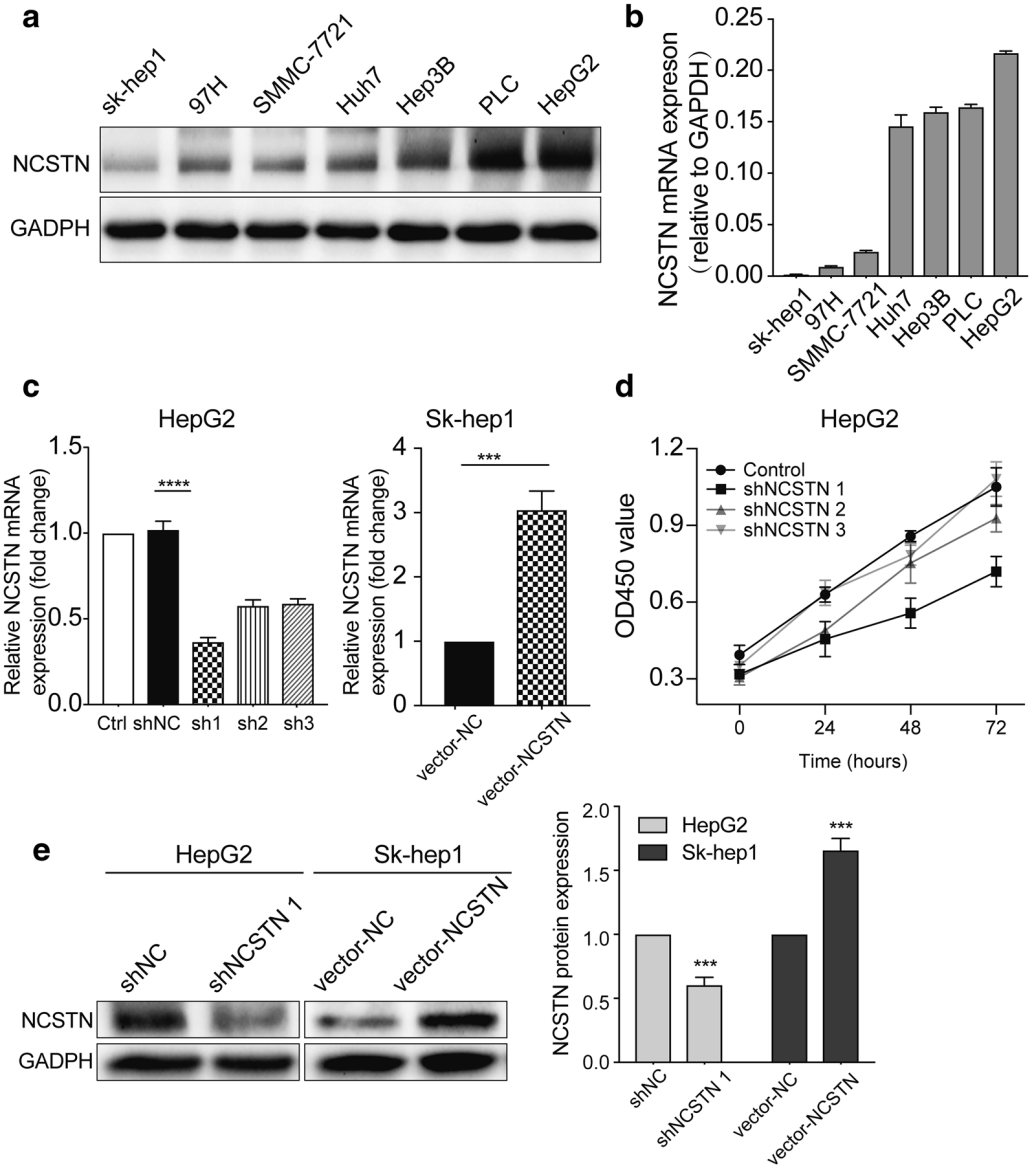
Knockdown efficiency of the NCSTN gene in HepG2 and Sk-hep1 cells. **a** NCSTN protein expression in various HCC cell lines. **b** mRNA expression of NCSTN in seven HCC cell lines. **c** qRT-PCR analysis of the knockdown efficiency of NCSTN in HepG2 cells and the overexpression efficiency of NCSTN in Sk-hep1 cells. **d** Different cell growth rates after treatment with shNCSTN 1, shNCSTN 2 and shNCSTN 3. **e** Western blotting analysis of the knockdown efficiency of NCSTN in HepG2 cells and the overexpression efficiency of NCSTN in Sk-hep1 cells. The data represent the mean ± SD of three independent experiments. **P < 0.01, ***P < 0.001, compared with siNC

To confirm the lowest and highest expression levels of NCSTN in 7 different HCC cell lines, we used Western blotting and qRT-PCR to detect the protein and mRNA expression levels of NCSTN, respectively. Figure 3a shows that NCSTN protein expression was lowest in Sk-hep1 cells and highest in HepG2 cells among the 7 HCC cell lines. Similar results were obtained from qRT-PCR (Fig. 3b). However, not all mRNA and protein expression levels were consistent among the 7 HCC cell lines. We found that Sk-hep1 cells expressed lower NCSTN than the other 6 cell lines, while HepG2 cells expressed higher NCSTN than the other cells (Fig. 3b). To determine the most efficient RNAi sequence in HepG2 cells, we measured NCSTN mRNA expression after knocking down the NCSTN gene with 3 different shRNA sequences (Fig. 3c). This result showed that all 3 shRNA sequences could knock down NCSTN mRNA expression, but shNCSTN 1 dramatically inhibited NCSTN expression in HepG2 cells. We also conducted a cell growth experiment to estimate which shRNA sequences could affect HCC cell viability. As shown in Fig. 3d, shNCSTN 1 could reduce many more viable cells than the other two shRNA sequences. To further verify the knockdown efficiency of shNCSTN 1, we measured the protein expression level of NCSTN using Western blotting (Fig. 3e). The shNCSTN 1 sequence markedly downregulated NCSTN protein expression in HepG2 cells. Thus, these results suggested that the RNAi efficiency was adequate. On the other hand, for gain-of-function analyses, we overexpressed NCSTN in Sk-hep1 cells, which have relatively low NCSTN expression. The NCSTN expression level was significantly increased in NCSTN-transfected Sk-hep1 cells, as assessed by qRT-PCR and Western blotting (Fig. 3c, e).

### NCSTN downregulation inhibited HepG2 cell proliferation, and NCSTN-transfected Sk-hep1 cells showed higher proliferative ability

**Fig. 4:**
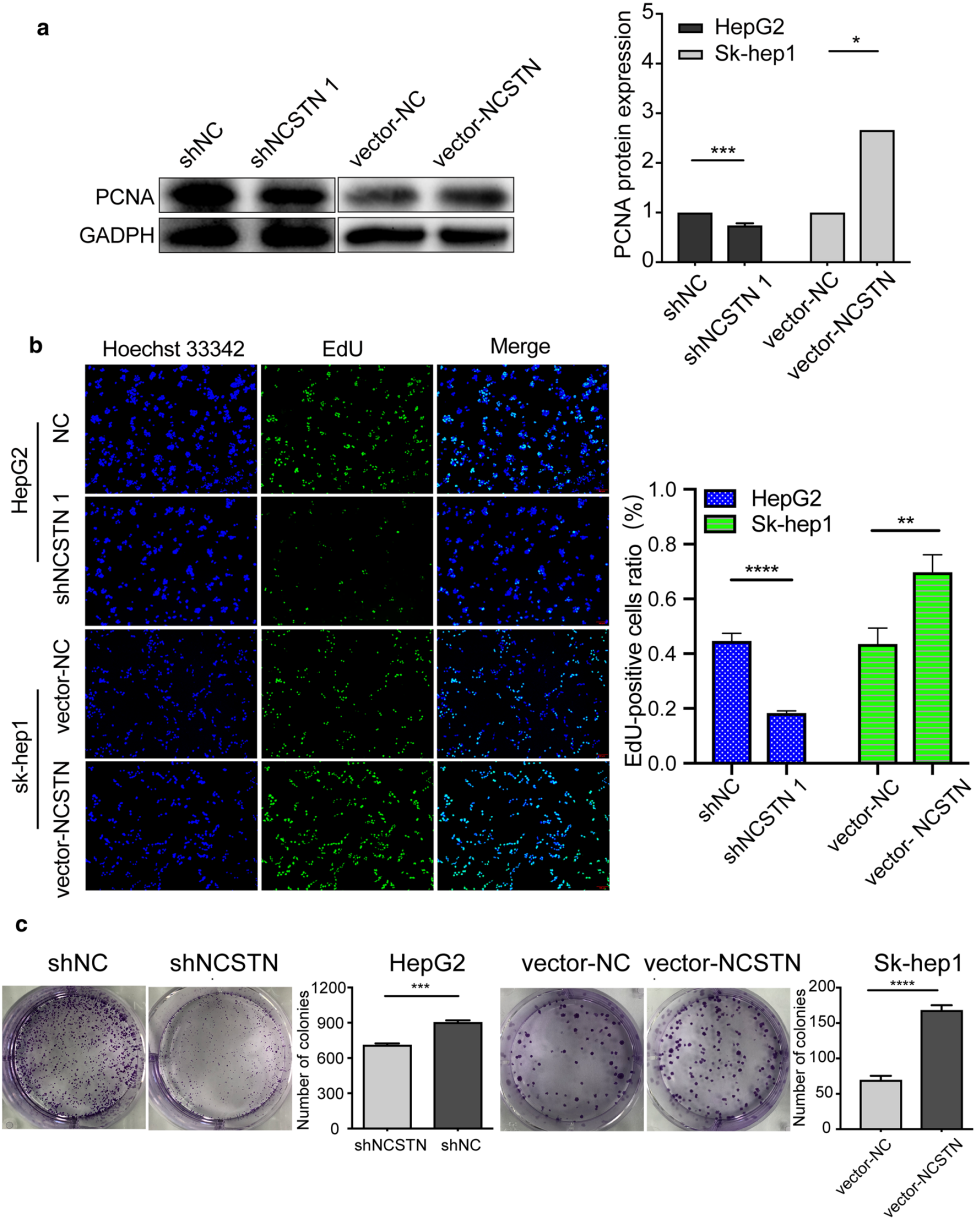
Regulation of cell proliferation by NCSTN in liver cancer cells. **a**, **b** Western blotting showing PCNA protein expression in HepG2 and Sk-hep1 cells. **c**, **d** Cell proliferation was detected with a 5-ethynyl-2-deoxyuridine (EdU) assay in HCC cells after NCSTN silencing. **e** Cell proliferation ability was detected by colony formation assay. *P < 0.05, **P < 0.01, ***P < 0.001

Provided that the correlation between NCSTN and cell proliferation was ambiguous, Western blotting analysis was adopted to identify PCNA expression. As shown in Fig. 4a, after NCSTN silencing in HepG2 cells, the shNCSTN 1 group exhibited markedly decreased PCNA expression compared with the shNC group. This result indicated that NCSTN knockdown had an inhibitory effect on PCNA, which could promote the process of leading strand synthesis during DNA replication. Since NCSTN expression was related to PCNA expression, we then determined proliferation in HepG2 cells with NCSTN knockdown by using EdU thymidine analogue incorporation and clone formation assays (Fig. 4b, c). Consistent with the Western blotting results, the EdU-positive cell ratio was much lower in the shNCSTN 1 group than in the other groups, and the number of colonies was lower in the shNCSTN 1 group than in the shNC group.

On the other hand, relatively contrary results were obtained in the gain-of-function assay. These data suggested that NCSTN regulated proliferation in both HepG2 and Sk-hep1 cells.

### NCSTN expression affected apoptosis in both HepG2 and Sk-hep1 cells

**Fig. 5:**
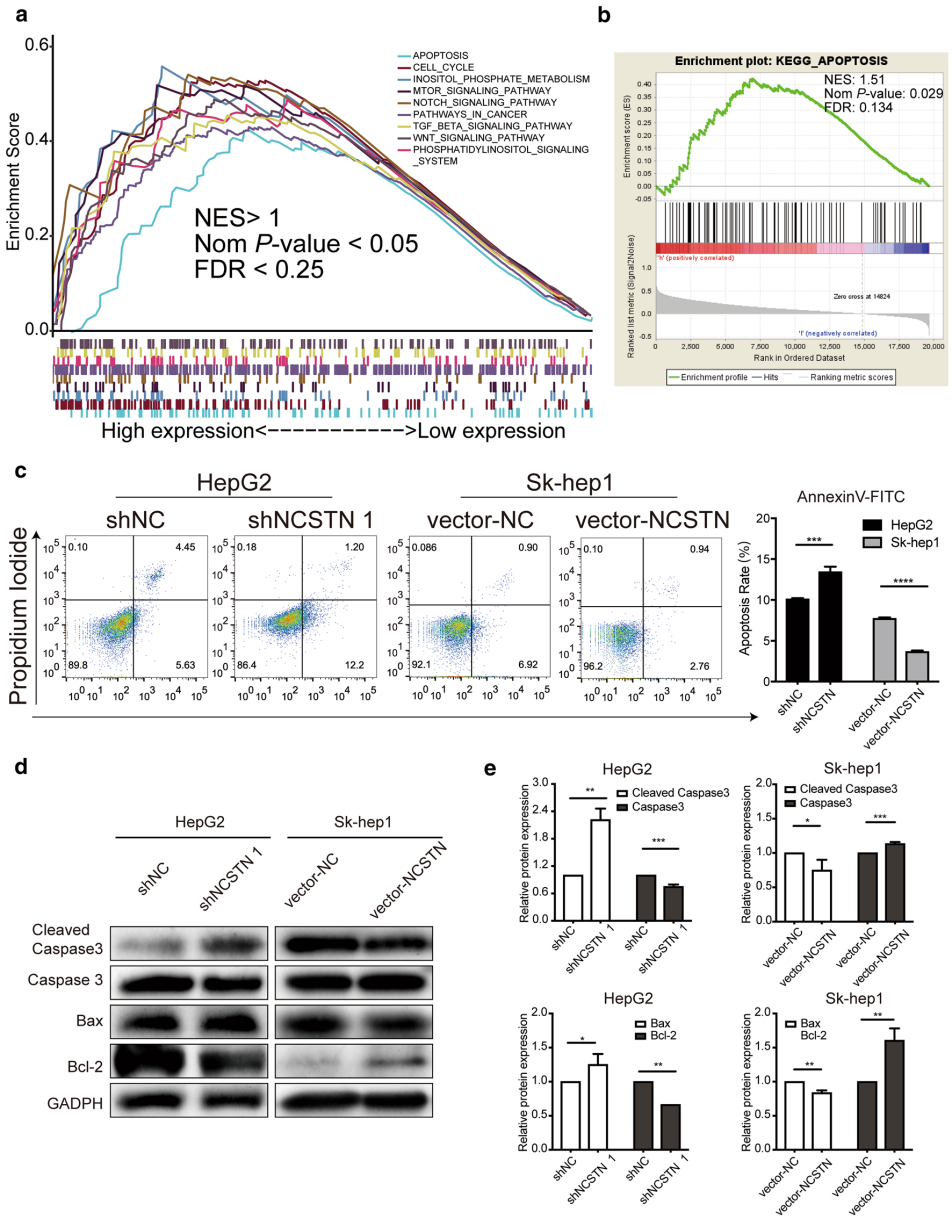
Depletion of NCSTN induced cell apoptosis. **a**, **b** Enriched pathways based on GSEA software. The *P*-value of each pathway was < 0.05, and the FDR was < 0.25. **c** Flow cytometry was used to study the correlation of NCSTN and apoptosis. **d** Protein expression of apoptotic markers analysed by Western blotting. *P < 0.05, **P < 0.01, ***P < 0.001

Based on the GSEA results for TCGA data, we found that 54 signalling pathways, including cancer-related pathways and apoptosis pathways (Fig. 5a, b), were enriched in the high NCSTN expression group (Additional file 4: Table S3). Considering the significance of NCSTN in regulating cell proliferation, we further investigated the antiapoptotic effect of NCSTN. Western blotting analysis revealed that the protein expression of cleaved caspase 3 and Bax was upregulated, while caspase 3 and Bcl-2 were downregulated in the shNCSTN 1 group. Comparably, cleaved caspase 3 and Bax were downregulated, and caspase 3 and Bcl-2 were upregulated the vector-NCSTN group (Fig. 5d, e). Moreover, Annexin V staining and flow cytometry were used to further study whether NCSTN and apoptosis were strongly related. As shown in Fig. 5c, the rate of apoptotic HCC cells in the shNCSTN 1 group was significantly different from that in the control group. A much higher early apoptosis rate appeared in the vector-NC group than in the vector-NCSTN group. Therefore, we concluded that HCC cell line apoptosis was strongly related to NCSTN expression.

### NCSTN regulated cell proliferation and apoptosis through the PI3K/Akt pathway

**Fig. 6:**
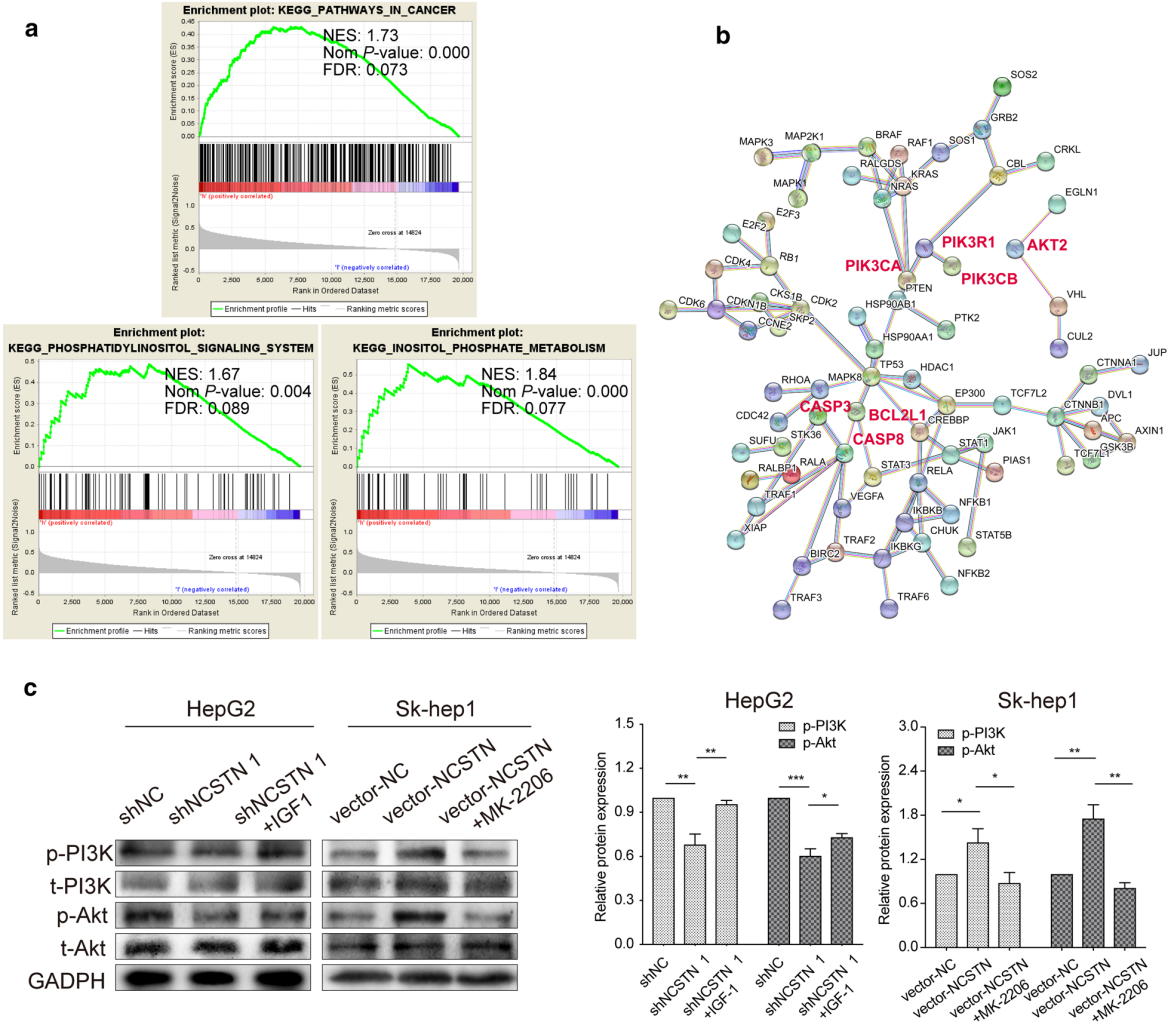
NCSTN exerts its functions via PI3K/Akt activation. **a** GSEA-enriched pathways. The *P*-value was lower than 0.05, and the FDR was lower than 0.25. **b** Enriched genes of cancer-related pathways. A protein–protein network was constructed using the String database (https://string-db.org). The confidence of the interaction score is greater than 0.990, and the disconnected nodes are hidden. **c** The phosphorylation levels of the PI3K/Akt signalling pathway were detected by Western blotting. t-PI3K, t-Akt and GAPDH were used as internal references

In general, most oncogenes promote cancer progression via specific signalling pathways. As exhibited in Fig. 6a, KEGG results for GSEA contained two PI3K-related pathways: “INOSITOL_PHOSPHATE_METABOLISM” and “PHOSPHATIDYLINOSITOL_SIGNALING_SYSTEM”. PIK3CA, PIK3R1, PIK3CB and AKT2 all participated in the “PATHWAYS_IN_CANCER” exhibited in the PPI network (Fig. 6b, Additional file 5: Table S4). Therefore, we further investigated the phosphorylation levels of PI3K and Akt in HCC cell lines treated with specific NCSTN-targeting shRNAs or NCSTN plasmids. As a result, p-PI3K and p-Akt levels were markedly decreased in the shNCSTN 1 group compared with the shNC group, while IGF1, an agonist of the PI3K/Akt pathway, reversed the effect that resulted from knocking down NCSTN. On the other hand, NCSTN overexpression significantly increased the phosphorylation levels of PI3K and Akt. MK-2206, an inhibitor of the PI3K/Akt pathway, suppressed the high expression of p-PI3K and p-Akt caused by upregulating NCSTN in Sk-hep1 cells (Fig. 6c). Therefore, we found that NCSTN might activate the PI3K/Akt pathway to regulate cell proliferation and inhibit apoptosis in HCC.

### Lentivirus-mediated NCSTN silencing in HepG2 cells inhibited tumour proliferation in vivo

**Fig. 7:**
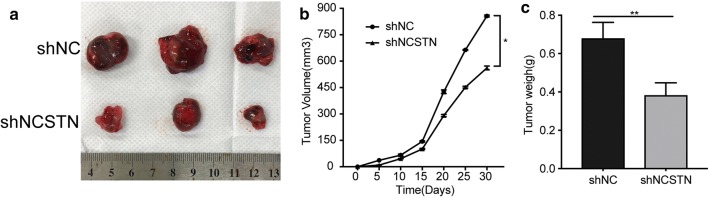
Antitumour effect of shNCSTN against HCC in vivo. **a** Mice were injected with HepG2 or shNCSTN-HepG2 cells. The photograph shows the dissected tumours from each group. **b** Tumour volume was measured every 5 days, and tumour growth curves were created for each group. **c** The dissected tumour weights indicated that shNCSTN mildly suppressed the proliferation of subcutaneous tumours

Evidence from the cell lines and human tissues mentioned above indicated that NCSTN is implicated in HCC tumourigenesis. To further confirm the hepatocarcinogenesis of NCSTN in vivo, xenograft tumour mouse models were injected with HepG2 cells. Compared with that in the shNC group treated with vector-NC plasmid, tumour growth in the group treated with vector-NCSTN plasmid was significantly inhibited (Fig. 7a, b). In addition, the tumour weight in the shNCSTN group was 57.01% of that of the shNC group at the end of the study (Fig. 7c). In summary, we concluded that NCSTN also possessed antitumour effects in vivo.

## Discussion

In our present study, we found that NCSTN promotes cell growth and negatively regulates apoptosis via the PI3K/Akt signalling pathway. Indeed, 53 out of 60 HCC patients in our centre had higher NCSTN expression in tumour specimens than in paired normal tissues. Furthermore, by regulating the PI3K/Akt pathway, NCSTN depletion in HepG2 cells and NCSTN overexpression in Sk-hep1 cells both had obviously different effects on cell proliferation and antiapoptosis processes, hallmarks of carcinogenesis and programmed cell death.

Previous studies have shown that the γ-secretase complex, comprised of PS-1, APH-1, PEN-2 and NCSTN, is recognized as a therapeutic option for treating HCC [10, 20–22]. However, inhibitors of the γ-secretase complex are well known to have serious adverse effects, as they are not cell selective or system specific [23]. Targeted therapy strategies have been developed and are now widely used in clinics. To date, several monoclonal antibodies against NCSTN have been developed and have shown great efficacy in inhibiting cancer cell proliferation in vitro and in vivo [23]. However, whether NCSTN monoclonal antibodies could effectively act on liver cancer cells remains unclear. Therefore, it is pivotal to determine the specific function of NCSTN in HCC.

NCSTN, the largest constituent of the γ-secretase complex [24], has a single transmembrane domain and a large extracellular domain with functional regions [8]. Intriguingly, it has been reported that NCSTN could exert its function alone rather than participate in the γ-secretase complex in mouse muscle [25], which indicates that NCSTN is likely to exert some functions independently. Similarly, in light of our bioinformatics analysis results, we found that NCSTN is overexpressed in HCC over 9 microarray datasets from the Oncomine database. However, the expression levels of PS-1, APH-1 and PEN-2 are slightly higher in HCC tissues than in normal tissues in only several microarray datasets. According to the analysis of TCGA data with GEPIA, we further confirmed that the mRNA expression of PS-1, APH-1 and PEN-2 in HCC patients from TCGA was not significantly different between tumour specimens and normal tissue specimens. On the other hand, we also found that among TCGA data for HCC patients analysed by KMPlotter, only NCSTN expression was closely related to survival time, not PS-1, APH-1 and PEN-2. We also found that NCSTN was altered most among these four genes in TCGA-LIHC patients according to the corresponding CNV and mutation data analysed by the CBioPortal database. Together, we hypothesized that the tumourigenesis of NCSTN can also play a role without the involvement of other members of the γ-secretase complex. The bioinformatics analysis results prompted us to explore the independent function of NCSTN in HCC patients.

Next, we investigated the mRNA and protein expression of NCSTN in patients at our centre by using qRT-PCR and IHC staining. The IHC score results for 60 HCC patients showed high degrees of consistency with those obtained from qRT-PCR. It turned out that high NCSTN expression and poor prognosis are closely related. Likewise, Lee et al. [12] also identified NCSTN as an oncogene that is upregulated and frequently amplified in human HCC. In all, although its correlation with the clinicopathological features of HCC patients failed to have great significance for both TCGA data (Additional file 2: Table S1) and 60 patients from our centre (Additional file 1: Table S2), NCSTN expression was closely related to the clinical prognosis of HCC patients. These observations led us to test the role of NCSTN in tumour cell proliferation and apoptosis. Therefore, we analysed the impact of NCSTN inhibition in HepG2 cells using transient shRNA oligos targeting NCSTN mRNA and NCSTN overexpression in Sk-hep1 cells using an NCSTN plasmid.

Other than its requirement for the γ-secretase complex, NCSTN is likely to regulate cell death through the PI3K/Akt and P53/caspase3 pathways in fibroblasts, human cells and neurons [26]. Furthermore, Lombardo et al. [6] reported that NCSTN regulates Akt activation in breast cancer cells and that NCSTN inhibition in malignant breast cells can inhibit breast tumour formation in vivo. Studies have also demonstrated that anti-NCSTN monoclonal antibodies exert antitumour effects in invasive breast cancer cells [8]. Notably, these results are consistent with not only the KEGG pathway results for GSEA based on TCGA data but also our observation that NCSTN depletion could induce apoptosis and reduce proliferation in HCC cell lines, partly through the PI3K/Akt pathway.

However, these conclusions are based on the responses of only 2 HCC cell lines and several nude mice, so they might not reflect the process of intact organisms in the human body. The precise oncogenic mechanism of NCSTN needs to be further elucidated in detail.

## Conclusions

Collectively, our findings support the hypothesis that NCSTN modulates proliferation and apoptosis through the PI3K/Akt pathway, thereby causing tumourigenesis in HCC. Our present work provides a better understanding of the potentially oncogenic mechanism of NCSTN in silico, in vitro and in vivo. We proposed that NCSTN, independent of the GS complex, may be a promising target for HCC treatment development.

## Supplementary information

**Additional file 1: Table S2.** Correlation between NCSTN expression and clinicopathological characteristics of 60 HCC patients in our center.

**Additional file 2: Table S1.** Relationship between NCSTN expression and clinicopathological characteristics in 370 HCC patients from TCGA database.

**Additional file 3: Figure S1.** mRNA Expression level of APH-1, PEN-2 and PS-1 in 60 patients.

**Additional file 4: Table S3.** Gene sets enriched in phenotype high.

**Additional file 5: Table S4.** The enriched genes of cancer-related pathways.

## Data Availability

Not applicable.

## Abbreviations

GS complex: γ-Secretase complex
HCC: Hepatocellular carcinoma
OS: Overall survival
DFS: Disease-free survival
GSEA: Gene set enrichment analysis
CNV: Copy number variation
NCSTN: Nicastrin
PS-1: Presenilin-1
APH-1: Anterior-pharynx defective-1
PEN-2: PSEN enhancer 2
