# Supplementary material for: Effect of nicastrin on hepatocellular carcinoma proliferation and apoptosis through PI3K/AKT signalling pathway modulation
Source: Cancer Cell Int. 2020 Mar 24;20:91. doi: 10.1186/s12935-020-01172-4 (PMC7092570; doi:10.1186/s12935-020-01172-4)
Supplement: Supplementary file 1 — Additional file 1: Table S2. Correlation between NCSTN expression and clinicopathological characteristics of 60 HCC patients in our center. [file 12935_2020_1172_MOESM1_ESM.docx]

Table S2. Correlation between NCSTN expression and clinicopathological characteristics of 60 HCC patients in our center.

| clinicopathological characteristics | NCSTN expression | | *P*-value | Adjusted x2 |
| --- | --- | --- | --- | --- |
|  | Low (30) | High (30) |  |  |
| Age（years） |  |  | 0.778159686 | 0.079365079 |
| ≤50 | 10 | 8 |  |  |
| >50 | 20 | 22 |  |  |
| Gender |  |  | 0.666954529 | 0.185185185 |
| Male | 26 | 28 |  |  |
| Female | 4 | 2 |  |  |
| hepatitis virus infection |  |  | 0.332921608 | 0.9375 |
| HBsAg (+) | 26 | 22 |  |  |
| HBsAg (-) | 4 | 8 |  |  |
| Child-Pugh |  |  | 1 | 0 |
| A | 30 | 29 |  |  |
| B | 0 | 1 |  |  |
| C | 0 | 0 |  |  |
| Cirrhosis |  |  | 0.605376134 | 0.266963293 |
| Yes | 16 | 13 |  |  |
| No | 14 | 17 |  |  |
| AFP (ug/L) |  |  | 1 | 0 |
| ≤400 | 21 | 20 |  |  |
| >400 | 9 | 10 |  |  |
| TNM stage |  |  | 0.288184265 | 1.128084606 |
| T1+T2 | 14 | 9 |  |  |
| T3+T4 | 16 | 21 |  |  |
| Tumor size (cm) |  |  | 0.778159686 | 0.079365079 |
| ≤5 | 10 | 8 |  |  |
| >5 | 20 | 22 |  |  |
| Vascular invasion |  |  | 0.278957622 | 1.172161172 |
| Yes | 17 | 22 |  |  |
| No | 13 | 8 |  |  |
| Histological grade |  |  | 0.227 | 2.963 |
| G1 | 5 | 1 |  |  |
| G2 | 12 | 14 |  |  |
| G3 | 13 | 15 |  |  |

Notes: (a) The median expression level of NCSTN was used as the cutoff. Low NCSTN expression in each of the 30 patients was defined as a value below the 50th percentile. High NCSTN expression in each of the 18 patients was defined as a value above the 50th percentile. (b) χ2 test, ***P*< 0.01.
