# Supplementary material for: Effect of nicastrin on hepatocellular carcinoma proliferation and apoptosis through PI3K/AKT signalling pathway modulation
Source: Cancer Cell Int. 2020 Mar 24;20:91. doi: 10.1186/s12935-020-01172-4 (PMC7092570; doi:10.1186/s12935-020-01172-4)
Supplement: Supplementary file 2 — Additional file 2: Table S1. Relationship between NCSTN expression and clinicopathological characteristics in 370 HCC patients from TCGA database. [file 12935_2020_1172_MOESM2_ESM.docx]

Table S1. Relationship between NCSTN expression and clinicopathological characteristics in 370 HCC patients from TCGA database.

| clinicopathological characteristics | NCSTN expression | | *P*-value | Adjusted x^2^ |
| --- | --- | --- | --- | --- |
|  | Low (185) | High (185) |  |  |
| Age (years) |  |  | 0.898579066 | 0.01624517 |
| ≤50 | 40 | 38 |  |  |
| >50 | 145 | 147 |  |  |
| Gender |  |  | 0.183580724 | 1.768395898 |
| Male | 131 | 118 |  |  |
| Female | 54 | 67 |  |  |
| hepatitis virus infection |  |  | 0.54635801 | 0.36388223 |
| HBV/HCV | 23 | 28 |  |  |
| No | 162 | 157 |  |  |
| Child-Pugh |  |  | 0.196 | 4.687 |
| A | 116 | 100 |  |  |
| B | 11 | 10 |  |  |
| C | 1 | 0 |  |  |
| No | 57 | 75 |  |  |
| Cirrhosis |  |  | 1 | 0 |
| Yes | 39 | 39 |  |  |
| No | 146 | 146 |  |  |
| AFP (ug/L) |  |  | 0.213971798 | 1.544347826 |
| ≤400 | 176 | 169 |  |  |
| >400 | 9 | 16 |  |  |
| TNM stage |  |  | 0.041248409 | 4.165780288 |
| I+II | 142 | 114 |  |  |
| III+IV | 38 | 52 |  |  |
| not known | 5 | 19 |  |  |
| Histological grade |  |  | 0.405 | 2.917 |
| G1 | 32 | 28 |  |  |
| G2 | 93 | 84 |  |  |
| G3 | 53 | 68 |  |  |
| G4 | 7 | 5 |  |  |

Notes: (a) The median expression level of NCSTN was used as the cutoff. Low VSIG4 expression in each of the 185 patients was defined as a value below the 50th percentile. High NCSTN expression in each of the 185 patients was defined as a value above the 50th percentile. (b) χ2 test, *P<0.05, **P<0.01.

Abbreviations: HBV, hepatitis B virus; HCC, hepatocellular carcinoma; HCV, hepatitis C virus; TCGA, The Cancer Genome Atlas.
