# Supplementary figures and images for: Effect of nicastrin on hepatocellular carcinoma proliferation and apoptosis through PI3K/AKT signalling pathway modulation
Source: Cancer Cell Int. 2020 Mar 24;20:91. doi: 10.1186/s12935-020-01172-4 (PMC7092570; doi:10.1186/s12935-020-01172-4)

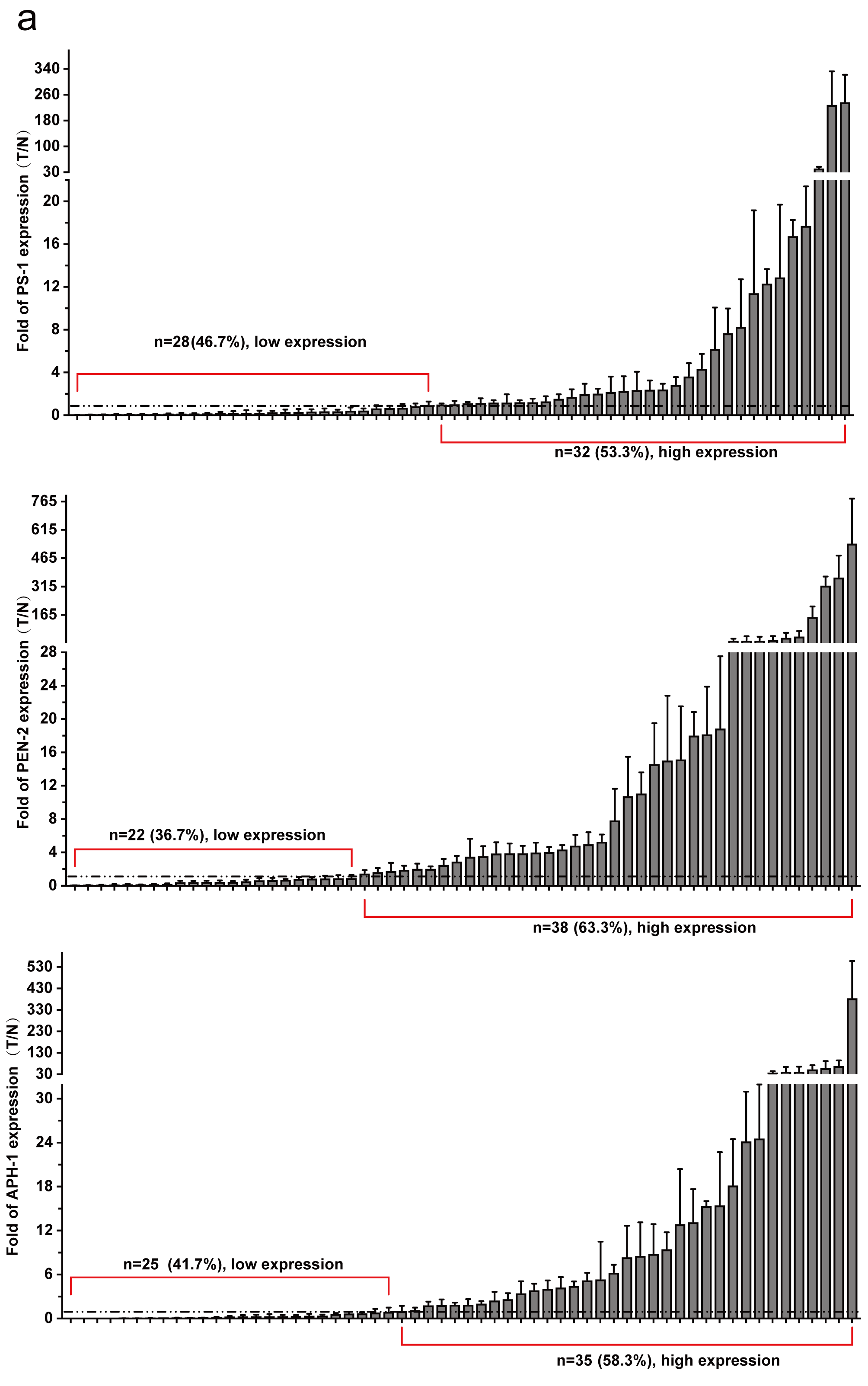

Supplement: Supplementary file 3 — Additional file 3: Figure S1. mRNA Expression level of APH-1, PEN-2 and PS-1 in 60 patients. [file 12935_2020_1172_MOESM3_ESM.tif]
