# Supplementary material for: Effect of nicastrin on hepatocellular carcinoma proliferation and apoptosis through PI3K/AKT signalling pathway modulation
Source: Cancer Cell Int. 2020 Mar 24;20:91. doi: 10.1186/s12935-020-01172-4 (PMC7092570; doi:10.1186/s12935-020-01172-4)
Supplement: Supplementary file 4 — Additional file 4: Table S3. Gene sets enriched in phenotype high. [file 12935_2020_1172_MOESM4_ESM.docx]

Table S3. Gene sets enriched in phenotype high.

| NAME | NES | NOM *P*-val | FDR q-val |
| --- | --- | --- | --- |
| KEGG_UBIQUITIN_MEDIATED_PROTEOLYSIS | 2.109293 | 0 | 0.016033694 |
| KEGG_GLYCOSYLPHOSPHATIDYLINOSITOL_GPI_ANCHOR_BIOSYNTHESIS | 1.8909321 | 0 | 0.15647231 |
| KEGG_ERBB_SIGNALING_PATHWAY | 1.8805145 | 0 | 0.1153778 |
| KEGG_WNT_SIGNALING_PATHWAY | 1.8799667 | 0 | 0.08792738 |
| KEGG_NEUROTROPHIN_SIGNALING_PATHWAY | 1.8481609 | 0 | 0.08297089 |
| KEGG_INOSITOL_PHOSPHATE_METABOLISM | 1.8413978 | 0 | 0.077271596 |
| KEGG_OOCYTE_MEIOSIS | 1.8314595 | 0 | 0.06576068 |
| KEGG_ENDOCYTOSIS | 1.7599151 | 0 | 0.08243473 |
| KEGG_MTOR_SIGNALING_PATHWAY | 1.7592041 | 0 | 0.07747909 |
| KEGG_CHRONIC_MYELOID_LEUKEMIA | 1.7564123 | 0 | 0.07038905 |
| KEGG_PATHWAYS_IN_CANCER | 1.7331736 | 0 | 0.07316031 |
| KEGG_NOTCH_SIGNALING_PATHWAY | 1.7228897 | 0 | 0.07332404 |
| KEGG_THYROID_CANCER | 1.798442 | 0.001988072 | 0.07735888 |
| KEGG_COLORECTAL_CANCER | 1.7313266 | 0.002004008 | 0.07127 |
| KEGG_PROSTATE_CANCER | 1.6620376 | 0.00203252 | 0.084634714 |
| KEGG_RNA_DEGRADATION | 1.8356252 | 0.00204918 | 0.07149484 |
| KEGG_PROGESTERONE_MEDIATED_OOCYTE_MATURATION | 1.6821355 | 0.00204918 | 0.09108182 |
| KEGG_BASAL_TRANSCRIPTION_FACTORS | 1.8728801 | 0.002079002 | 0.077733554 |
| KEGG_INSULIN_SIGNALING_PATHWAY | 1.6672595 | 0.003853565 | 0.08404841 |
| KEGG_TIGHT_JUNCTION | 1.672751 | 0.004 | 0.090145074 |
| KEGG_ADHERENS_JUNCTION | 1.7954596 | 0.004065041 | 0.07227963 |
| KEGG_GNRH_SIGNALING_PATHWAY | 1.5850365 | 0.00407332 | 0.10118795 |
| KEGG_PHOSPHATIDYLINOSITOL_SIGNALING_SYSTEM | 1.6691395 | 0.004081633 | 0.08919366 |
| KEGG_RIG_I_LIKE_RECEPTOR_SIGNALING_PATHWAY | 1.7090087 | 0.004149378 | 0.07838572 |
| KEGG_SMALL_CELL_LUNG_CANCER | 1.7808324 | 0.005870841 | 0.07651662 |
| KEGG_ENDOMETRIAL_CANCER | 1.7406081 | 0.005940594 | 0.07704191 |
| KEGG_LONG_TERM_POTENTIATION | 1.6742728 | 0.006 | 0.09276721 |
| KEGG_MELANOGENESIS | 1.6032258 | 0.007736944 | 0.1041862 |
| KEGG_ACUTE_MYELOID_LEUKEMIA | 1.6355976 | 0.0078125 | 0.08590958 |
| KEGG_MAPK_SIGNALING_PATHWAY | 1.5396144 | 0.007968128 | 0.11815372 |
| KEGG_GLIOMA | 1.5890485 | 0.008080808 | 0.101065986 |
| KEGG_NON_SMALL_CELL_LUNG_CANCER | 1.6560286 | 0.00814664 | 0.08082305 |
| KEGG_PANCREATIC_CANCER | 1.656996 | 0.010162601 | 0.08277501 |
| KEGG_EPITHELIAL_CELL_SIGNALING_IN_HELICOBACTER_PYLORI_INFECTION | 1.6260899 | 0.014314928 | 0.090356305 |
| KEGG_CELL_CYCLE | 1.7589598 | 0.014705882 | 0.07270241 |
| KEGG_N_GLYCAN_BIOSYNTHESIS | 1.6679766 | 0.015686275 | 0.086638786 |
| KEGG_NUCLEOTIDE_EXCISION_REPAIR | 1.8107842 | 0.01632653 | 0.07525304 |
| KEGG_SPLICEOSOME | 1.7352878 | 0.01927195 | 0.075660914 |
| KEGG_REGULATION_OF_AUTOPHAGY | 1.6578766 | 0.019607844 | 0.08482626 |
| KEGG_TGF_BETA_SIGNALING_PATHWAY | 1.5989312 | 0.022132797 | 0.10491517 |
| KEGG_RENAL_CELL_CARCINOMA | 1.5658848 | 0.023622047 | 0.1094431 |
| KEGG_VASOPRESSIN_REGULATED_WATER_REABSORPTION | 1.5933251 | 0.027888447 | 0.100885995 |
| KEGG_APOPTOSIS | 1.5057871 | 0.028688524 | 0.13392454 |
| KEGG_REGULATION_OF_ACTIN_CYTOSKELETON | 1.5453038 | 0.0332681 | 0.116647296 |
| KEGG_CYTOSOLIC_DNA_SENSING_PATHWAY | 1.5982077 | 0.033898305 | 0.10034762 |
| KEGG_PURINE_METABOLISM | 1.5114758 | 0.035789475 | 0.13176988 |
| KEGG_BASAL_CELL_CARCINOMA | 1.4876523 | 0.035928145 | 0.1365167 |
| KEGG_SPHINGOLIPID_METABOLISM | 1.5707506 | 0.038022812 | 0.108565465 |
| KEGG_RIBOFLAVIN_METABOLISM | 1.5333593 | 0.039337475 | 0.11812193 |
| KEGG_LONG_TERM_DEPRESSION | 1.4465069 | 0.03937008 | 0.1544981 |
| KEGG_T_CELL_RECEPTOR_SIGNALING_PATHWAY | 1.6398095 | 0.04016064 | 0.08582526 |
| KEGG_PYRIMIDINE_METABOLISM | 1.5530094 | 0.041666668 | 0.11621759 |
| KEGG_FC_EPSILON_RI_SIGNALING_PATHWAY | 1.4992137 | 0.04715128 | 0.13652568 |
| KEGG_MISMATCH_REPAIR | 1.6405973 | 0.049792532 | 0.087859586 |

NES: normalized enrichment score; NOM: nominal; FDR: false discovery rate. Gene sets with NOM *P*-value< 0.05 and FDR q-value> 0.25 are considered as significant.
