# Supplementary material for: Effect of nicastrin on hepatocellular carcinoma proliferation and apoptosis through PI3K/AKT signalling pathway modulation
Source: Cancer Cell Int. 2020 Mar 24;20:91. doi: 10.1186/s12935-020-01172-4 (PMC7092570; doi:10.1186/s12935-020-01172-4)
Supplement: Supplementary file 5 — Additional file 5: Table S4. The enriched genes of cancer-related pathways. [file 12935_2020_1172_MOESM5_ESM.docx]

Table S4. The enriched genes of cancer-related pathways.

| NAME | PROBE | RANK IN GENE LIST | RANK METRIC SCORE | RUNNING ES | CORE ENRICHMENT |
| --- | --- | --- | --- | --- | --- |
| row_0 | TPR | 8 | 0.665076792 | 0.012337341 | Yes |
| row_1 | LAMC1 | 113 | 0.516169071 | 0.0168518 | Yes |
| row_2 | TPM3 | 127 | 0.509358108 | 0.025944844 | Yes |
| row_3 | CRKL | 135 | 0.504892826 | 0.03526277 | Yes |
| row_4 | BRAF | 174 | 0.48826009 | 0.04265758 | Yes |
| row_5 | MAPK1 | 181 | 0.486597896 | 0.051676493 | Yes |
| row_6 | PIAS1 | 222 | 0.474697858 | 0.058707777 | Yes |
| row_7 | MAPK9 | 297 | 0.459845632 | 0.063694835 | Yes |
| row_8 | EP300 | 304 | 0.458473533 | 0.07217453 | Yes |
| row_9 | SMAD3 | 365 | 0.449220479 | 0.07768236 | Yes |
| row_10 | ABL1 | 398 | 0.44462803 | 0.084551126 | Yes |
| row_11 | DVL3 | 408 | 0.443542063 | 0.09258929 | Yes |
| row_12 | MSH2 | 437 | 0.437966347 | 0.09953732 | Yes |
| row_13 | ARNT | 452 | 0.4359743 | 0.10717165 | Yes |
| row_14 | CREBBP | 465 | 0.434519142 | 0.114881575 | Yes |
| row_15 | PIAS3 | 504 | 0.429023981 | 0.12114067 | Yes |
| row_16 | CUL2 | 507 | 0.428459108 | 0.1292519 | Yes |
| row_17 | SMAD4 | 630 | 0.413837999 | 0.1308729 | Yes |
| row_18 | RELA | 654 | 0.412174106 | 0.13758518 | Yes |
| row_19 | SMAD2 | 671 | 0.410787702 | 0.14463311 | Yes |
| row_20 | E2F3 | 702 | 0.407516181 | 0.15089384 | Yes |
| row_21 | VHL | 829 | 0.396984756 | 0.15198472 | Yes |
| row_22 | TRAF6 | 869 | 0.393859565 | 0.15751787 | Yes |
| row_23 | RALBP1 | 884 | 0.393133402 | 0.16433083 | Yes |
| row_24 | STK4 | 1025 | 0.381295472 | 0.16439642 | Yes |
| row_25 | STK36 | 1034 | 0.380847961 | 0.17128432 | Yes |
| row_26 | MSH6 | 1090 | 0.377233833 | 0.17567071 | Yes |
| row_27 | TCF7L2 | 1142 | 0.373233378 | 0.1801874 | Yes |
| row_28 | CTNNB1 | 1172 | 0.37173456 | 0.18581384 | Yes |
| row_29 | STAT5B | 1343 | 0.360983342 | 0.18393753 | Yes |
| row_30 | MAPK8 | 1351 | 0.360446692 | 0.19048603 | Yes |
| row_31 | ITGA6 | 1425 | 0.356131226 | 0.19353636 | Yes |
| row_32 | CDK2 | 1452 | 0.354460746 | 0.19898686 | Yes |
| row_33 | GSK3B | 1513 | 0.350702882 | 0.20260584 | Yes |
| row_34 | XIAP | 1514 | 0.35064593 | 0.20932868 | Yes |
| row_35 | PRKCA | 1666 | 0.342009366 | 0.20807181 | Yes |
| row_36 | BIRC2 | 1670 | 0.34178859 | 0.21446958 | Yes |
| row_37 | PIK3CA | 1675 | 0.341520518 | 0.22081046 | Yes |
| row_38 | FZD5 | 1810 | 0.334902853 | 0.22029708 | Yes |
| row_39 | DVL2 | 1883 | 0.330300689 | 0.2229039 | Yes |
| row_40 | CTBP1 | 2009 | 0.32364437 | 0.22264041 | Yes |
| row_41 | SUFU | 2107 | 0.318762094 | 0.22373228 | Yes |
| row_42 | ITGB1 | 2128 | 0.317583919 | 0.22878624 | Yes |
| row_43 | FGFR3 | 2163 | 0.316031635 | 0.23308598 | Yes |
| row_44 | CTNNA1 | 2213 | 0.313548207 | 0.23656183 | Yes |
| row_45 | EGLN1 | 2272 | 0.311103016 | 0.23952508 | Yes |
| row_46 | PIAS2 | 2275 | 0.310978055 | 0.24538387 | Yes |
| row_47 | IKBKB | 2286 | 0.310352653 | 0.2508167 | Yes |
| row_48 | NCOA4 | 2306 | 0.309590727 | 0.25576916 | Yes |
| row_49 | SKP2 | 2314 | 0.309163183 | 0.26133442 | Yes |
| row_50 | KRAS | 2326 | 0.308387935 | 0.2666778 | Yes |
| row_51 | NRAS | 2327 | 0.308374405 | 0.2725902 | Yes |
| row_52 | APPL1 | 2343 | 0.307675481 | 0.27771294 | Yes |
| row_53 | ITGA2 | 2393 | 0.305037111 | 0.28102562 | Yes |
| row_54 | BRCA2 | 2482 | 0.301145494 | 0.28224546 | Yes |
| row_55 | CHUK | 2508 | 0.2999973 | 0.2867035 | Yes |
| row_56 | APC | 2517 | 0.299586952 | 0.2920334 | Yes |
| row_57 | CCDC6 | 2523 | 0.299272686 | 0.29751253 | Yes |
| row_58 | RXRB | 2655 | 0.294500619 | 0.29637977 | Yes |
| row_59 | TFG | 2659 | 0.294352978 | 0.30186808 | Yes |
| row_60 | CASP8 | 2679 | 0.293295175 | 0.30650812 | Yes |
| row_61 | HSP90AB1 | 2785 | 0.28999725 | 0.3066345 | Yes |
| row_62 | GRB2 | 2929 | 0.283914179 | 0.30467778 | Yes |
| row_63 | PIAS4 | 2931 | 0.283816636 | 0.31006756 | Yes |
| row_64 | PTCH1 | 3015 | 0.279990166 | 0.31114057 | Yes |
| row_65 | SOS2 | 3093 | 0.276626855 | 0.3124596 | Yes |
| row_66 | CBL | 3120 | 0.275817186 | 0.31640226 | Yes |
| row_67 | JUP | 3122 | 0.275729418 | 0.321637 | Yes |
| row_68 | PTK2 | 3148 | 0.27473405 | 0.32561067 | Yes |
| row_69 | RARA | 3152 | 0.274660796 | 0.3307214 | Yes |
| row_70 | DAPK1 | 3191 | 0.273527026 | 0.33399922 | Yes |
| row_71 | VEGFA | 3207 | 0.273023903 | 0.33845758 | Yes |
| row_72 | CCNE2 | 3288 | 0.269872487 | 0.33949184 | Yes |
| row_73 | JAK1 | 3366 | 0.266606212 | 0.34061873 | Yes |
| row_74 | RAF1 | 3490 | 0.261440128 | 0.33926612 | Yes |
| row_75 | PLCG1 | 3499 | 0.261063576 | 0.3438574 | Yes |
| row_76 | TCF7 | 3549 | 0.259295613 | 0.34629312 | Yes |
| row_77 | TRAF5 | 3554 | 0.259080172 | 0.3510534 | Yes |
| row_78 | HDAC2 | 3574 | 0.2584171 | 0.35502473 | Yes |
| row_79 | BCL2L1 | 3601 | 0.257418543 | 0.35861465 | Yes |
| row_80 | PIK3CB | 3686 | 0.253723443 | 0.3591323 | Yes |
| row_81 | CEBPA | 3774 | 0.250820577 | 0.35943905 | Yes |
| row_82 | SOS1 | 3775 | 0.25057283 | 0.3642432 | Yes |
| row_83 | TRAF2 | 3809 | 0.24923709 | 0.36731404 | Yes |
| row_84 | AXIN1 | 3856 | 0.247637108 | 0.36968145 | Yes |
| row_85 | RALA | 3878 | 0.246787056 | 0.3733263 | Yes |
| row_86 | CDK4 | 4050 | 0.239767 | 0.3690742 | Yes |
| row_87 | LAMA3 | 4142 | 0.23703301 | 0.3689096 | Yes |
| row_88 | TP53 | 4151 | 0.236686796 | 0.37303352 | Yes |
| row_89 | NFKB1 | 4186 | 0.235423252 | 0.37578776 | Yes |
| row_90 | CASP3 | 4226 | 0.234121725 | 0.3782583 | Yes |
| row_91 | FZD3 | 4238 | 0.233633697 | 0.38216844 | Yes |
| row_92 | ITGAV | 4273 | 0.232439905 | 0.3848655 | Yes |
| row_93 | LAMA5 | 4304 | 0.231218129 | 0.3877461 | Yes |
| row_94 | PPARD | 4312 | 0.23083128 | 0.39180952 | Yes |
| row_95 | MLH1 | 4336 | 0.229962215 | 0.3950283 | Yes |
| row_96 | CDK6 | 4393 | 0.228169531 | 0.39650497 | Yes |
| row_97 | HDAC1 | 4512 | 0.223925799 | 0.39469185 | Yes |
| row_98 | ARNT2 | 4520 | 0.223454326 | 0.3986138 | Yes |
| row_99 | E2F2 | 4618 | 0.21994321 | 0.39781106 | Yes |
| row_100 | TRAF1 | 4626 | 0.219770998 | 0.40166244 | Yes |
| row_101 | CKS1B | 4647 | 0.218893975 | 0.40482426 | Yes |
| row_102 | SMO | 4713 | 0.216013923 | 0.40560213 | Yes |
| row_103 | RHOA | 4715 | 0.21599637 | 0.40969163 | Yes |
| row_104 | FZD6 | 4744 | 0.215129584 | 0.41236725 | Yes |
| row_105 | RB1 | 4856 | 0.211282372 | 0.41067398 | Yes |
| row_106 | CBLB | 5000 | 0.206344455 | 0.40723002 | Yes |
| row_107 | MAPK3 | 5005 | 0.206196219 | 0.41097638 | Yes |
| row_108 | PTEN | 5087 | 0.203661367 | 0.41068944 | Yes |
| row_109 | APC2 | 5129 | 0.202466026 | 0.41244954 | Yes |
| row_110 | IGF1R | 5268 | 0.197967231 | 0.40910375 | Yes |
| row_111 | FGFR2 | 5298 | 0.197127223 | 0.4113825 | Yes |
| row_112 | WNT3 | 5431 | 0.192366019 | 0.40823978 | Yes |
| row_113 | BCR | 5455 | 0.191732317 | 0.4107256 | Yes |
| row_114 | CDC42 | 5460 | 0.191566944 | 0.41419145 | Yes |
| row_115 | KITLG | 5461 | 0.1915344 | 0.4178637 | Yes |
| row_116 | TGFBR2 | 5490 | 0.190501958 | 0.42006716 | Yes |
| row_117 | FN1 | 5547 | 0.188376978 | 0.4207809 | Yes |
| row_118 | TCF7L1 | 5600 | 0.186776802 | 0.42167097 | Yes |
| row_119 | MSH3 | 5604 | 0.186588258 | 0.4250931 | Yes |
| row_120 | DVL1 | 5610 | 0.186478227 | 0.42840967 | Yes |
| row_121 | MAP2K1 | 5698 | 0.183435574 | 0.42742446 | Yes |
| row_122 | RALGDS | 5800 | 0.180574343 | 0.4256599 | Yes |
| row_123 | FGF13 | 5948 | 0.176711068 | 0.4214408 | Yes |
| row_124 | TRAF3 | 6151 | 0.17115818 | 0.41426906 | Yes |
| row_125 | RAD51 | 6152 | 0.171157613 | 0.41755062 | Yes |
| row_126 | RASSF5 | 6164 | 0.170654714 | 0.42025328 | Yes |
| row_127 | BMP4 | 6173 | 0.1704254 | 0.42310682 | Yes |
| row_128 | STAT1 | 6272 | 0.167649105 | 0.4212497 | Yes |
| row_129 | LAMB1 | 6472 | 0.162033722 | 0.41405824 | Yes |
| row_130 | NFKB2 | 6547 | 0.160213366 | 0.41330054 | Yes |
| row_131 | HSP90AA1 | 6584 | 0.158995703 | 0.41448596 | Yes |
| row_132 | PDGFB | 6620 | 0.158008829 | 0.4157042 | Yes |
| row_133 | PLD1 | 6664 | 0.157040164 | 0.41648987 | Yes |
| row_134 | TGFBR1 | 6697 | 0.156197608 | 0.41782862 | Yes |
| row_135 | PIK3R1 | 6740 | 0.155415311 | 0.4186349 | Yes |
| row_136 | AKT2 | 6751 | 0.155194342 | 0.4210929 | Yes |
| row_137 | CDKN1B | 6808 | 0.15371494 | 0.4211421 | Yes |
| row_138 | STAT3 | 6832 | 0.153222293 | 0.42288956 | Yes |
| row_139 | RXRG | 6912 | 0.150969431 | 0.42169586 | Yes |
| row_140 | FGF11 | 6914 | 0.150935605 | 0.42453796 | Yes |
| row_141 | TRAF4 | 6924 | 0.150730416 | 0.42696214 | Yes |
| row_142 | IKBKG | 6930 | 0.150622189 | 0.42959124 | Yes |
